# Lactic Acid Bacteria Surface Proteins in the Mechanisms of Cell Adhesion and Immunoregulation

**DOI:** 10.1002/fsn3.4517

**Published:** 2024-10-30

**Authors:** Ziheng Meng, Xianqing Huang, Mingwu Qiao, Lianjun Song, Yufei Liu, Dan Hai

**Affiliations:** ^1^ College of Food Science and Technology Henan Agricultural University Zhengzhou China; ^2^ Henan Engineering Technology Research Center of Food Processing and Circulation Safety Control Zhengzhou China

**Keywords:** cell adhesion, immunomodulatory molecules, immunoregulation, lactic acid bacteria, surface proteins

## Abstract

This study delves into the role of lactic acid bacteria (LAB) surface proteins in cell adhesion and immunoregulation. Using fluorescence microscopy, we observed distinct adhesion patterns on various cell types. LAB surface proteins demonstrated concentration‐dependent inhibition of Salmonella adhesion, with LAB69 exhibiting potent antagonistic effects. Genetic expression analysis revealed nuanced responses in key genes (MD2, TLR4, IL‐10, MUC3, MIF) across different cell types, highlighting the diverse immunomodulatory effects of LAB surface proteins. Modulation of pro‐inflammatory (TNF‐α) and anti‐inflammatory (IL‐10) cytokines further emphasized the complex interplay. In conclusion, this study underscores the pivotal role of LAB surface proteins in mediating cell adhesion and immunoregulation, providing a foundation for isolating specific immunomodulatory molecules within LAB surface proteins for potential applications in microbial ecological agents.

## Graphical abstract

Our manuscript explores the pivotal role of lactic acid bacteria surface proteins in mediating cell adhesion and immunoregulatory responses. Through intricate molecular interactions, these proteins facilitate crucial communication between lactic acid bacteria and host cells, modulating immune system function and highlighting their potential in functional food applications.

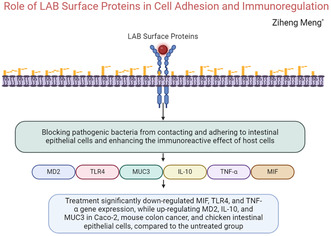

## 1 Introduction

As microbiota exploration progresses, lactic acid bacteria (LAB) have emerged as vital probiotics, crucial for intestinal microbial balance and host health. Surface proteins, particularly surface‐layer proteins of LAB, have garnered significant interest as potential bioactive molecules among researchers (Han et al. 2019). These proteins, integral to LAB cell surfaces, possess diverse structures and functionalities, believed to interact with host cells (Chen et al. 2018). Cell adhesion, a key step in microbe–host interaction, directly influences microbe survival and colonization in the host's intestinal tract. Previous studies suggest that LAB surface proteins can hinder the adhesion process of pathogenic bacteria (Zhang et al. 2013), reducing their invasion into the intestinal mucosa through competitive adhesion. Experimental results demonstrate concentration‐dependent inhibition of Salmonella adhesion upon the addition of LAB surface proteins, validated through various methods like fluorescence microscopy and plate counting.

Moreover, the influence of LAB surface proteins on immunoregulation has garnered considerable attention. Cytokines, pivotal in immune regulation, are specifically focused in this study, including factors like MD2, TLR4, IL‐10, MUC3, MIF, and TNF‐α. The results indicate significant regulation of these factors by LAB surface proteins across various cell types, elucidating their potential mechanisms in immunoregulation. For instance, upregulation of MD2 gene expression is observed in different cell lines, particularly significant after the addition of surface proteins in LAB35 and LAB69 (Qin, Wang, and Huang 2015). However, the TLR4 gene expression shows a complex regulatory pattern, with LAB35 exhibiting significant regulatory effects, whereas LAB69 and standard strain LG exhibit a downregulation trend.

The experimental design investigates the interaction between LAB strains (LAB35, LAB69, LG) and different cell types (Caco‐2 cells, mouse colon cancer cells, chicken small intestinal mucosal cells) to analyze cytokine expression changes. Special attention is paid to the influence of LAB surface proteins on Salmonella, exploring their regulatory effects on cytokine expression, IL‐10, and key factors like MUC3 (Fang et al. 2015).

## 2 Materials and Methods

### 2.1 Reagents and Equipment

#### 2.1.1 Strains, Cells, and Culture Media

The strains used in this study include LAB35 (*Lactobacillus salinus* strain HO 66), LAB69 (*Lactobacillus* sp. wx213), L76 (*Lactobacillus salinus* strain L13), and the standard strain LG (*L*. *rhamnosus* G, FSMM22).

Caco‐2 cells were procured from Shanghai Bogo Biotechnology Co., Ltd. (Shanghai, China); mouse colon cancer cells and chicken small intestinal mucosal epithelial cells were obtained from Wuhan Puno Life Technology Co., Ltd. (Wuhan, China).

The liquid MRS medium was prepared by weighing 20 g of glucose, 10 g of beef extract, 5 g of peptone, 5 g of anhydrous sodium acetate, 2 g of potassium dihydrogen phosphate, 0.25 g of manganese sulfate monohydrate, 0.58 g of magnesium sulfate heptahydrate, 2 g of diammonium hydrogen citrate, and adding 1 mL of Tween 80 to dissolve in 1 L of distilled water. The mixture was sterilized at 115°C for 30 min.

The solid MRS medium was prepared similarly to the liquid MRS medium, with the addition of 2% (w/v) agar powder.

Complete DMEM medium was composed of 90% DMEM culture medium, 10% fetal bovine serum, and 1% antibiotic‐antimycotic solution (penicillin and streptomycin).

Incomplete DMEM medium was prepared with 90% DMEM culture medium and 10% fetal bovine serum.

#### 2.1.2 Reagents

DMEM culture medium (Hyclone SH30022) and RPMI1640 culture medium (Hyclone SH30809) were obtained from Beijing Solabio Biotechnology Co., Ltd. Fetal bovine serum (FBS, BI 04‐001‐1 ACS) was purchased from Zhengzhou Hengmu Biotechnology Co., Ltd. The culture medium for chicken small intestinal mucosal epithelial cells was acquired from Wuhan Puno Life Technology Co., Ltd. Other reagents, including agar powder, methanol, ethanol, glycerol, isopropanol, glacial acetic acid, β‐mercaptoethanol, ammonium persulfate (APS), sodium dodecyl sulfate (SDS), acrylamide (Arc), bisacrylamide (Bis), glycine, Tris, Coomassie Brilliant Blue 250, and bromophenol blue, EDTA‐2Na, were purchased from Shanghai Maclyn Biotechnology Co., Ltd. The PAGE gel protein microrecovery kit and total RNA rapid extraction kit (including DNaseI) were obtained from Beijing Bomai De Biotechnology Co., Ltd. Lithium chloride was sourced from Shanghai Solabio Biotechnology Co., Ltd. The BCA protein assay kit was purchased from Biyun Tian Biotechnology Research Institute. The 5× loading buffer was obtained from Beijing Biyun Tian Biotechnology Research Institute. SDS‐PAGE standard protein (10–250 kDa) was purchased from Thermo Fisher Scientific. Dialysis bags (with molecular weight cutoffs of 3500 and 14,000 Da) were acquired from Shanghai Solabio Biotechnology Co., Ltd. The FITC fluorescence labeling kit and standard protein solution were prepared by dissolving γ‐globulin or bovine serum albumin (BSA) to prepare 1.0 and 0.1 mg/mL standard protein solutions. Coomassie Brilliant Blue G‐250 dye reagent was prepared by weighing 100 mg of Coomassie Brilliant Blue G‐250, dissolving it in 50 mL of 95% ethanol, and then adding 120 mL of 85% phosphoric acid, diluted with water to a total volume of 1 L. Hieff8qPCR SYBR Green Master Mix (High Rox Plus) was purchased from Shanghai Yisheng Biotechnology Co., Ltd. Hifairst Strand cDNA Synthesis SuperMix for qPCR (gDNA digester plus) was also obtained from Shanghai Yisheng Biotechnology Co., Ltd.

#### 2.1.3 Instruments

The instruments used in this study include the ST16 centrifuge (Thermo Fisher, USA), YS100 high‐pressure steam sterilizer (Beijing Faenke Trading Co., Ltd.), SW‐CJ‐1FD vertical laminar flow clean bench (Sujing Group Antai Corporation), PB2002‐N electronic balance with a sensitivity of 0.1 g (Mettler‐Toledo, Switzerland), Biofuge Primo R tabletop high‐speed refrigerated centrifuge (Heraeus, Germany), HWR constant temperature incubator at 36°C ± 1°C (Ningbo Jiangnan Instrument Co., Ltd.), 721E visible spectrophotometer (Shanghai Precision Instrument Co., Ltd.), Bench Top Pro vacuum freeze dryer (SP Scientific, USA), Nanodrop ND‐2000 (Thermo Fisher), fluorescence quantitative PCR instrument (ABI, USA), optical microscope DMLB model (Leica, Germany), upright fluorescence microscope DM6B (Leica), 1300 series II A2 type biological safety cabinet (Thermo Fisher), and J‐715 circular dichroism spectrometer (JASCO, Japan).

### 2.2 Methods

#### 2.2.1 Preparation of Lactic Acid Bacteria Suspension and Surface Protein‐Removed Lactic Acid Bacteria

Activated lactic acid bacteria LAB35, LAB69, and LG were inoculated in MRS liquid culture medium at a seeding volume of 1% (V/V) and cultured at 37°C for 18–24 h. The bacterial cells were collected by centrifugation at 6000 × g, 4°C for 10 min. The bacterial cells were washed three times with sterile PBS buffer, resuspended in DMEM culture medium without antibiotics, and the OD600 of the bacterial suspension was adjusted to the required concentration. For LAB69, bacterial suspensions were prepared by diluting the culture at gradients of 1:2, 1:4, 1:8, 1:16, and 1:32. One milliliter of each diluted bacterial suspension was plated on a sterile agar medium, and the bacterial colonies were counted for each gradient. The OD values of different dilutions of bacterial suspensions at 600 nm were measured using a 721E visible spectrophotometer. The relationship between the standard bacterial count and OD values was established as a standard curve with the formula *y* = 201.55*x* − 19.66 and R2 = 0.9869. Lactic acid bacteria (LAB35, LAB69, LG) were inoculated into sterilized MRS liquid culture medium and incubated at 37°C for 24 h. Subsequently, streak activation was performed on MRS solid culture medium. Well‐formed bacterial colonies were selected and inoculated into MRS liquid culture medium, followed by static fermentation at 37°C for 18 h. The 18‐h fermentation liquid was then transferred to new MRS culture medium at a ratio of 3% and incubated at 37°C for 24 h. A 35 mL portion of the fermentation liquid was centrifuged at 4°C and 5000 rpm/min for 15 min. The supernatant was discarded, and the precipitate was washed three times with cold PBS buffer. The precipitate was mixed with 5 mL of 5 mol/L LiCl solution, incubated in an ice‐water bath for 30 min, and then centrifuged (10,000 rpm/min, 10 min, 4°C). The precipitate obtained after the removal of surface proteins was resuspended in PBS buffer and set aside.

#### 2.2.2 Cell Culture

Caco‐2, chicken small intestinal mucosal epithelial cells (CHI‐iCell), and mouse colon cancer cells (CT26.WT) were cultured in different cell culture media. When the cells reached a good growth status (70% confluence), they were passaged using 0.25% trypsin–EDTA. The cells were cultured in an adherent manner, and the culture medium was replaced every 2–3 days. Subculturing was carried out the next day, and adhesion experiments were performed after approximately 3–4 passages. Well‐grown cells were digested, diluted in incomplete culture medium (DMEM/1640/chicken small intestinal mucosal epithelial cell culture medium) to a concentration of ~5 × 10^5^ cells/mL, and then plated in a 12‐well plate. The plate was placed in a cell culture dish and incubated at 37°C in a CO_2_ incubator until the cells adhered to the wall. The culture medium was replenished to continue cultivation.

#### 2.2.3 Extraction of Surface Proteins From *Lactic Acid Bacteria*

Following the method by Lu (2015), surface proteins were initially coarsely extracted using 5 mol/L LiCl, followed by resolubilization with 1 mol/L LiCl to obtain purified surface proteins after centrifugation. The experimental procedure involved taking 35 mL of fermentation liquid in a 50 mL centrifuge tube, centrifuging at 4°C and 5000 rpm/min for 15 min in a refrigerated centrifuge, discarding the supernatant, washing the precipitate three times with cold PBS buffer, and discarding the supernatant. The precipitate was mixed with 5 mL of 5 mol/L LiCl solution, processed in a shaker at 37°C and 180 rpm/min for 60 min, followed by centrifugation (10,000 rpm/min, 10 min, 4°C). The supernatant was transferred to a dialysis bag, sealed, placed in a dialysis cup, and dialyzed against pure water at 4°C for 48 h, with the addition of silver nitrate to the external solution until no further precipitation occurred. After dialysis with PBS for 24 h, a dialysis bag with a cutoff of 12,000 Da was selected based on preliminary experiments. Stirring was conducted during the dialysis process, with a solution change every 6 h. The protein in the dialysis bag was resolubilized with 10 mL of 1 mol/L LiCl, stirred in an ice bath for 15 min, then centrifuged (12,000 rpm/min, 40°C) for 20 min to remove the supernatant. The precipitate was washed once with an appropriate amount of sterile double‐distilled water, and after centrifugation (12,000 rpm/min, 40°C) for 20 min, the resulting precipitate was the purified surface protein. The protein was further concentrated using polyethylene glycol for 24 h. The final purified surface protein extract was dissolved in PBS and stored at −20°C. The concentration of the extracted surface proteins from Lactic Acid Bacteria LAB69 was determined using the Bradford protein concentration assay kit, yielding final concentrations of 0.84, 1.59, and 2.61 g/L.

#### 2.2.4 Methods on the Adhesion Effect of Surface Proteins on Three Types of Cells

The extracted surface proteins from LAB were labeled using a FITC fluorescence labeling kit. The labeled proteins were then washed multiple times and filtered through ultrafiltration tubes to retain the FITC‐labeled proteins and assess the labeling efficiency. The adhesion of the proteins to cells was then evaluated.

In the first treatment, the control group (Group 1) consisted of the three cultured cell lines, which were observed and photographed using the confocal function of an upright fluorescence microscope. In the second treatment, Caco‐2 cells, mouse colon cancer cells, and chicken intestinal mucosal epithelial cells were cultured to a concentration of 6 × 10^5^ cells/mL. Then, 500 μL of the FITC‐labeled surface proteins were added, and the cells were observed under a confocal microscope (DM6 B upright fluorescence microscope, Leica) at a magnification of 630× to evaluate changes in fluorescence intensity (Table 1).

**TABLE 1:** Observation of surface protein adhesion to cells before and after fluorescent labeling.

Control group: cells	Experimental group: fluorescently labeled surface protein + cells
Caco‐2 cells	Fluorescently labeled surface protein‐b + Caco‐2 cells
Mouse colon cancer cells	Fluorescently labeled surface protein‐b + Mouse colon cancer cells
Chicken intestinal mucosal epithelial cells	Fluorescently labeled surface protein‐b + Chicken intestinal mucosal epithelial cells

#### 2.2.5 Cytokine Expression in Three Types of Cells Before and After Adding Surface Proteins to Lactobacillus

In the experiment, the control group (CK) involved incubating Caco‐2 cells, mouse colon cancer cells, and chicken intestinal mucosal cells with PBS for 2 h. After incubation, RNA was extracted, and the expression of cytokines was analyzed. Treatment Group 1 involved incubating the three types of cells (Caco‐2 cells, mouse colon cancer cells, and chicken intestinal mucosal cells) with Lactobacillus strains LAB35, LAB69, and LG for 2 h. RNA was then extracted, and cytokine expression was assessed. Treatment Group 2 involved incubating the cells with Lactobacillus strains LAB35, LAB69, and LG, to which surface proteins had been added, for 2 h. RNA was subsequently extracted and cytokine expression was analyzed.

For both the control and the treatment groups, RNA was extracted from the Caco‐2 cells, mouse colon cancer cells, and chicken small intestinal mucosal cells incubated with bacteria using an RNA extraction kit. The following mixture was prepared according to the Hifair 1st Strand cDNA Synthesis SuperMix for qPCR kit: 2 μL of 2 × gDNA wiper Mix, 500 ng of RNA template, and RNase‐free ddH_2_O to a total volume of 10 μL. The mixture was gently pipetted, incubated at 42°C for 2 min, yielding the first reaction mixture. According to the reverse transcription kit, RNA was reverse transcribed into cDNA using 2 μL of 5 × qRT SuperMix II and 8 μL of the first reaction mixture. The reaction conditions were 25°C for 10 min, 42°C for 30 min, and 85°C for 5 min, resulting in cDNA templates stored at −20°C for later use. Using the primers listed in Table 2, qPCR was performed.

**TABLE 2:** Primers and their sequences for detection of three cell genes.

Gene name	Primer sequence (5′–3′)	Fragment size (bp)
TLR4	F: AGTCTGAAATTGCTGAGCTCAAAT	225
	R: GCGACGTTAAGCCATGGAAG	
MD2	F: AGCTCTGAAGGGAGAGACTGT	138
	R: AGAGCATTTCTTCTGGGCTCC	
TNF	F: CCCATGTTGTAGCAAACCCTC	140
	R: TATCTCTCAGCTCCACGCCA	
MIF	F: CCGGACAGGGTCTACATCAA	194
	R: GCGAAGGTGGAGTTGTTCCA	
IL10	F: AAGACCCAGACATCAAGGCG	86
	R: CAGGGAAGAAATCGATGACAGC	
MUC3	F: TACCTCTTCCTGGCGTCT	208
	R: CGAGTTGTCCTGCGTGAT	

The qPCR reaction system had a total volume of 50 μL, including 2 μL of 2 × qPCR Master Mix, 1 μL each of forward and reverse primers (10 μM), 2 μL of cDNA template, and ddH2O to make up 50 μL. The qPCR amplification conditions were set as follows: 94°C for 30 s; 94°C for 20 s, 55°C for 30 s, repeated for 45 cycles, with signal detection at 55°C. The melting curve conditions were: 95°C for 0 s, 60°C for 15 s, and 95°C for 0 s, with continuous signal detection.

#### 2.2.6 Statistical Analysis

Each experiment was repeated three times. The results and data were analyzed using one‐way ANOVA with SPSS 16.0 software (SPSS Inc., Chicago, IL, USA). The results are presented as mean (M) ± standard deviation (SD). The mean values were compared using Duncan's multiple range test (*p* < 0.05).

## 3 Adhesion Study of Lactic Acid Bacteria and Surface Proteins to Three Types of Cells

### 3.1 Adhesion of Lactic Acid Bacteria to Cells Before and After Surface Protein Addition

The adhesion of lactic acid bacteria to three types of cells was observed under an optical microscope (DMLB‐type optical microscope, Leica) with a magnification of 400×. Caco‐2 cells were observed to be circular in shape, CT26 mouse colon cancer cells exhibited a polygonal morphology, and chicken small intestinal mucosal epithelial cells appeared elliptical. However, at high cell densities, irregular cell shapes were observed, and bacterial adhesion was found to influence cell morphology.

#### 3.1.1 Adhesion of LAB69 to Caco‐2 Cells Before and After Addition of Surface Proteins

**FIGURE 1:**
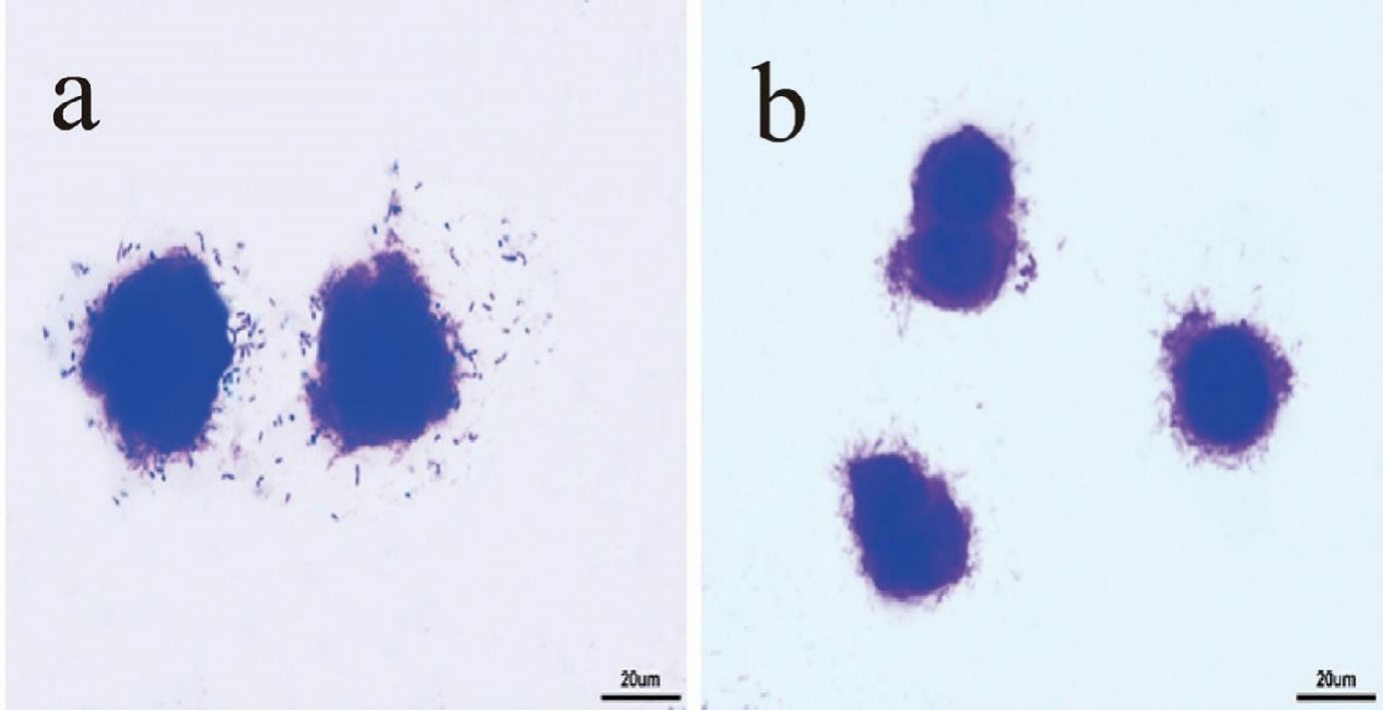
The results of light microscope experiment of Lactobacillus LAB69 adhering to Caco‐2 cells. (a) Results of LAB69 lactic acid bacteria adhering to Caco‐2 cells. (b) Results of LAB69 lactic acid bacteria + surface proteins adhering to Caco‐2 cells, magnification 400×.

As depicted in Figure 1, when LAB69 lactic acid bacteria were cultured alone with Caco‐2 cells, the bacteria prominently aggregated on the surface of Caco‐2 cells, with a high level of bacterial adhesion. However, after the addition of surface proteins, it was observed that the adhesion of lactic acid bacteria to Caco‐2 cells decreased.

#### 3.1.2 Adhesion of Lactic Acid Bacteria to Mouse Colon Cancer Cells Before and After Addition of Surface Proteins

**FIGURE 2:**
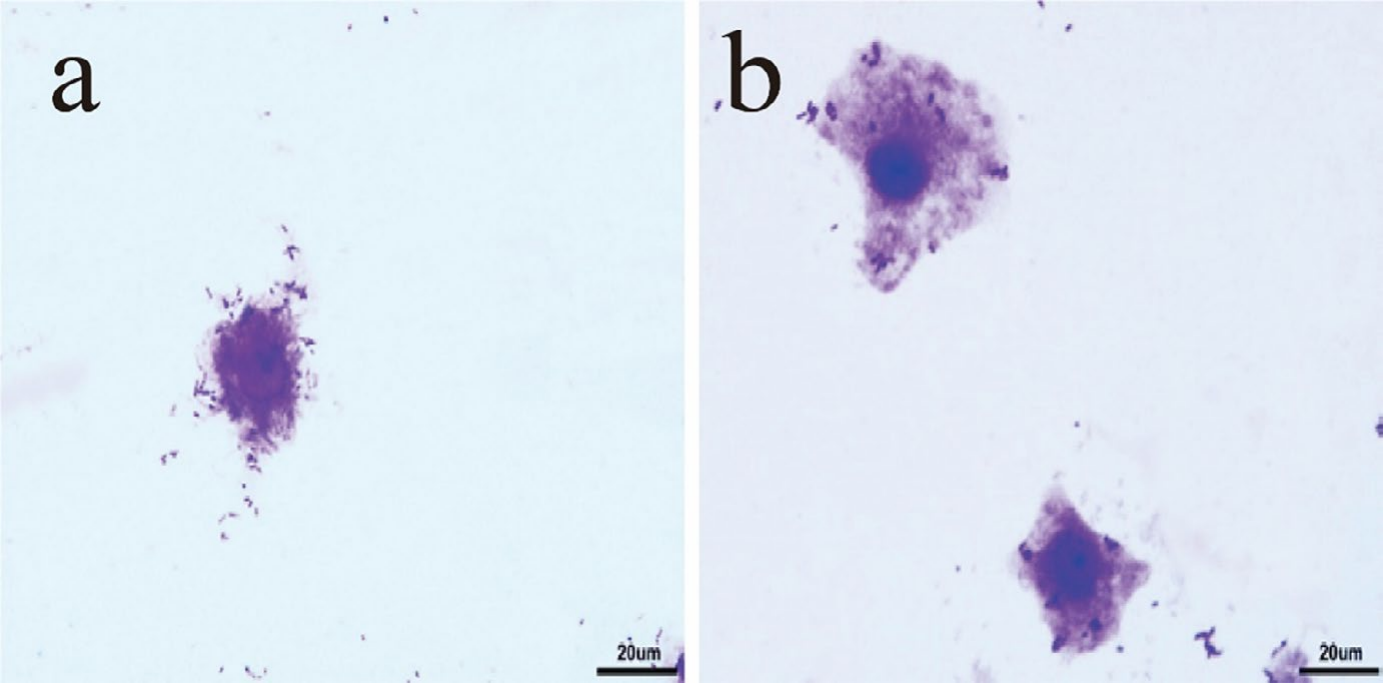
The results of light microscope experiment of Lactobacillus LAB69 adhering to CT26.WT cells. (a) Results of LAB69 lactic acid bacteria adhering to Caco‐2 cells. (b) Results of LAB69 lactic acid bacteria + surface proteins adhering to Caco‐2 cells, magnification 400×.

As observed in Figure 2, when LAB69 lactic acid bacteria were cultured alone with mouse colon cancer cells, the bacteria prominently aggregated on the surface of the cancer cells, exhibiting a high level of bacterial adhesion. However, after the addition of surface proteins, it was observed that the adhesion of lactic acid bacteria to mouse colon cancer cells decreased.

#### 3.1.3 Adhesion of Lactic Acid Bacteria to Chicken Small Intestinal Mucosal Epithelial Cells Before and After Addition of Surface Proteins

**FIGURE 3:**
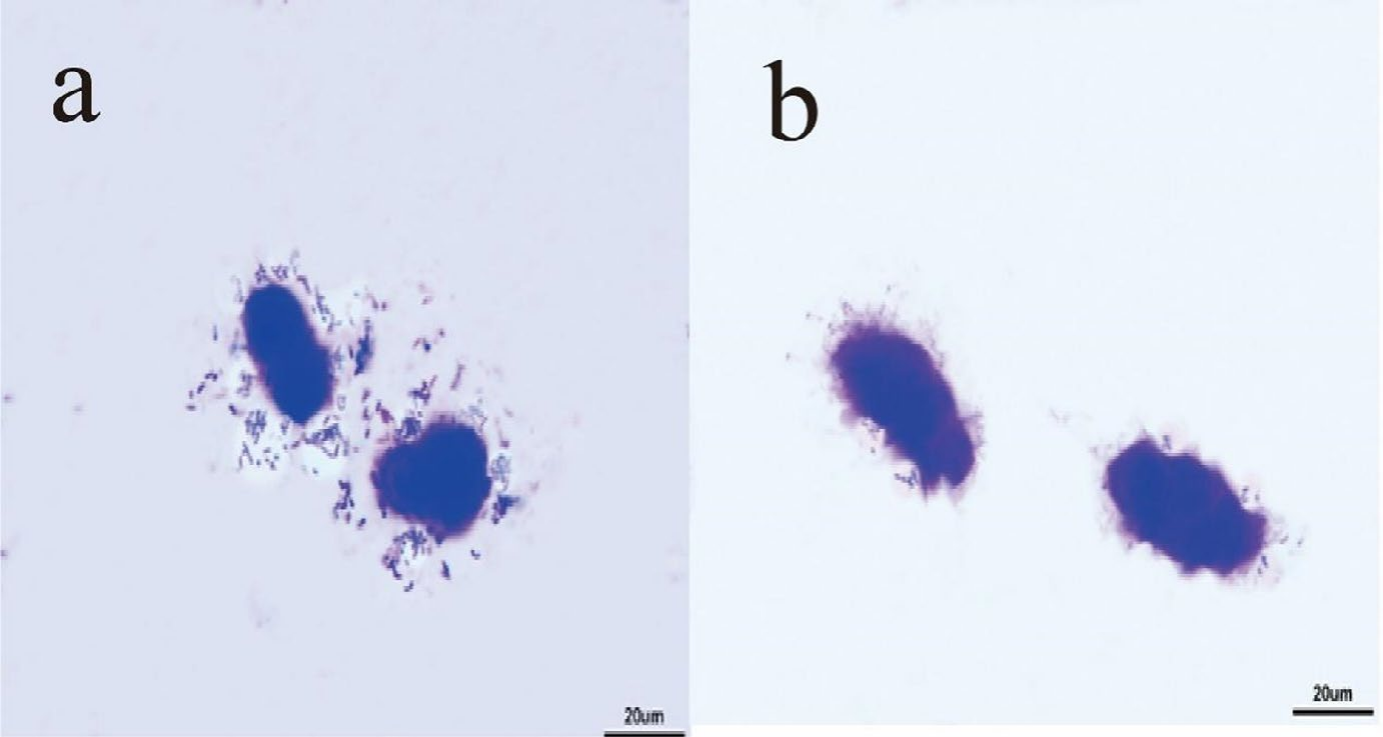
The results of light microscope experiment of Lactobacillus LAB69 adhering to epithelial cells of small intestine in chicken. (a) Results of LAB69 lactic acid bacteria adhering to Caco‐2 cells. (b) Results of LAB69 lactic acid bacteria + surface proteins adhering to Caco‐2 cells, magnification 400×.

As depicted in Figure 3, when LAB69 lactic acid bacteria were cultured alone with chicken small intestinal mucosal epithelial cells, the bacteria prominently aggregated on the cell surface, exhibiting a high level of adhesion. However, after the addition of surface proteins, it was observed that the adhesion of lactic acid bacteria to chicken small intestinal mucosal epithelial cells decreased.

### 3.2 Adhesion Index of Cells Before and After Addition of Lactic Acid Bacteria With Surface Proteins

**FIGURE 4:**
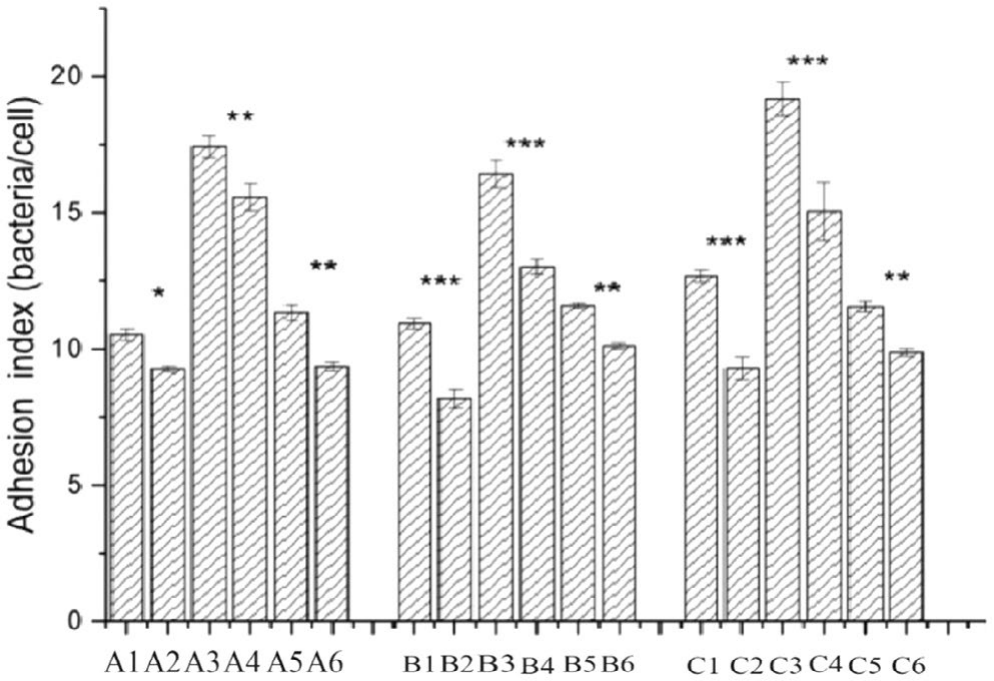
Adhesion index of the Lactobacillus to Caco‐2, mouse colon cancer cells and chicken intestinal epithelial cells. A1: LAB35‐Caco‐2; A2: LAB35 + S‐Caco‐2; A3: LAB69‐Caco‐2; A4: LAB69 + S‐Caco‐2; A5: LG‐Caco‐2; A6: LG + S‐Caco‐2; B1: LAB35‐CT26.WT; B2: LAB35 + S‐CT26.WT; B3: LAB69‐CT26.WT; B4: LAB69 + S‐CT26.WT; B5: LG‐CT26.WT; B6: LG + S‐CT26.WT; C1: LAB35‐CHI‐iCell; C2: LAB35 + S‐CHI‐iCell; C3: LAB69‐CHI‐iCell; C4: LAB69 + S‐CHI‐iCell; C5: LG‐ CHI‐iCell; C6: LG + S‐CHI‐iCell. LAB refers to the adhesion index of lactic acid bacteria alone to the three types of cells; LAB + S represents the adhesion index of lactic acid bacteria + surface proteins to the three types of cells.

Figures 1, 2, 3 show optical microscope comparison images of LAB35, LAB69, and LG lactic acid bacteria cultured alone with Caco‐2 cells, mouse colon cancer cells (CT26.WT), and chicken small intestinal mucosal epithelial cells, as well as images after lactic acid bacteria were cultured with the addition of surface proteins. Figure 4 depicts the adhesion index of cells before and after the addition of surface proteins (extracted from surface proteins of LAB69 lactic acid bacteria). Through these four figures, a decreasing trend in the adhesion ability of lactic acid bacteria to the three types of cells is evident after the addition of surface proteins. This suggests the possible presence of common ligands in the added surface proteins that bind to the three types of cells, blocking adhesion receptors on the cell surface and resulting in a decrease in the number of adhered bacteria.

Horizontal comparison reveals that when lactic acid bacteria were cultured alone with these three types of cells, the adhesion quantity of LAB69 lactic acid bacteria was significantly higher than that of LAB35 and standard strain LG. This may be related to the high content of surface proteins in LAB69 lactic acid bacteria, leading to strong adhesion ability. After the addition of surface proteins, the adhesion quantity of lactic acid bacteria to cells showed a decreasing trend.

Vertical comparison shows that the adhesion quantity of the three strains of bacteria to chicken small intestinal mucosal epithelial cells is higher than that to Caco‐2 cells and mouse colon cancer cells (CT26.WT). This may be attributed to the fact that these three strains of lactic acid bacteria were isolated from chicken intestines, showing better adhesion to chicken small intestinal mucosa. In summary, the adhesion index detected after lactic acid bacteria were cultured with the addition of surface proteins decreased, and there were differences between bacterial strains and different types of cells. The adhesion quantity of lactic acid bacteria decreased after the addition of surface proteins. Sun et al. (2012) confirmed with the same method that purified surface protein S1pA significantly reduced adhesion to cells, and S1pB significantly reduced the adhesion of *L*. *crispatus K313* to HT‐29 cells. This is consistent with our research results, indicating that purified surface proteins likely bind to cell surface receptors, occupy binding sites, and reduce the adhesion quantity of lactic acid bacteria, confirming the role of surface proteins in cell adhesion.

### 3.3 Detection of Cell Adhesion After Different Treatment of Surface Protein of Lactobacillus

Through Table 3, it is evident that LAB69‐A (1 × 10^2^ CFU/mL) represents a low concentration of lactic acid bacteria suspension, LAB69‐B (1 × 10^5^ CFU/mL) is a medium concentration lactic acid bacteria suspension, and LAB69‐C (1 × 10^8^ CFU/mL) is a high concentration lactic acid bacteria suspension. The differences in the detected quantity of lactic acid bacteria after the addition of different concentrations reveal that, overall, the high concentration lactic acid bacteria suspension exhibits greater adhesion to chicken intestinal mucosal cells. A longitudinal comparison of medium concentration lactic acid bacteria changes in Table 3 reveals that, compared to the control group, the first group, after the removal of surface proteins followed by their re‐addition, displays slightly lower adhesion of lactic acid bacteria to chicken intestinal mucosal cells than the control group (7.30 × l0^4^ CFU/mL compared to 1.70 × l0^5^ CFU/mL). The experiment found that after removing surface proteins, the addition of surface proteins increased the adhesion capability of lactic acid bacteria to cells, confirming the adhesive role of surface proteins and verifying the reversible binding ability of surface proteins to cell walls (Sára and Sleytr 2000). Chen et al. (2007a) incubated ST‐III bacterial bodies, from which surface proteins were removed, in the extracted surface protein solution overnight before conducting adhesion experiments. The results showed that the adhesion rate of ST‐III was restored to over 80%, close to untreated bacterial bodies (*p* > 0.05). This experiment also demonstrated through reversible binding experiments that surface proteins are involved in the adhesion process of lactic acid bacteria to chicken intestinal mucosal cells. In the second group, where lactic acid bacteria were treated with added surface proteins, the adhesion of lactic acid bacteria to chicken intestinal mucosal cells decreased to 5.0 × l0^3^ CFU/mL, two orders of magnitude lower than the control group. This is because the added surface proteins bind to receptor sites on the cell surface, preventing lactic acid bacteria from adhering to the cells. In the third group, lactic acid bacteria with removed surface proteins adhered to chicken intestinal mucosal cells, and it was found that a small number of lactic acid bacteria could still adhere to chicken intestinal mucosal cells, but the adhesion capability significantly decreased to 3.67 × l0^2^ CFU/mL, three orders of magnitude lower than the control group. The reduced adhesion capability of lactic acid bacteria to cells after the removal of surface proteins is attributed to the role of lactic acid bacteria's surface proteins in adhesion within the bacterial structure (Shimazu et al. 1999). Vicente et al. (2008) found in their 2008 experiment that lactic acid bacteria rich in surface proteins also had relatively high adhesion, and when surface proteins were removed, the adhesion of bacterial bodies significantly decreased. Our experimental results align with those of Vicente et al. Research also revealed that the adhesion capability of lactic acid bacteria to cells did not completely disappear. This is consistent with Anderson, Jürgens, and Nüsslein‐Volhard's (1985) findings in 1985 regarding the adhesion of bifidobacteria. They suggested that lipoteichoic acid (LTA) on the cell wall of bifidobacteria is associated with adhesion, indicating LTA's involvement in the adhesion of lactobacilli to intestinal epithelial cells. Schneitz, Nuotio, and Lounatma (2010) also considered that the surface carbohydrates of lactic acid bacteria are related to adhesion, as many lactic acid bacteria can produce extracellular polysaccharides (EPS), which may also participate in the adhesion process (Li, Ye, and Qian 2012).

**TABLE 3:** Adhesion of Lactobacillus to chicken small intestinal mucosal cells after removal of surface protein and addition of different concentrations of surface protein.

Treatment groups	LAB69‐A + b	LAB69‐B + b	LAB69‐B + b
Control group: lactic acid bacteria adherent cells	1.33 × l0^2^ ± 0.25	1.70 × l0^5^ ± 0.49	1.67 × l0^8^ ± 0.38
*Lactic acid bacteria* removing surface protein + surface protein adhering cells	0.92 × l0^2^ ± 0.33	7.30 × l0^4^ ± 0.49	5.30 × l0^7^ ± 0.26
*Lactic acid bacteria* + surface protein adherent cells	0.12 × l0^2^ ± 0.17	5.0 × l0^3^ ± 0.25	8.33 × l0^5^ ± 0.36
*Lactic acid bacteria* adherent cells removing surface proteins	0.03 × l0^2^ ± 0.21	3.67 × l0^2^ ± 0.37	4.0 × l0^4^ ± 0.32

*Note:* LAB69‐A concentration is 1 × 10^2^ CFU/mL, LAB69‐B concentration is 1 × 10^5^ CFU/mL, LAB69‐C concentration is 1 × 10^8^ CFU/mL, b is surface protein concentration is 1.59 g/L.

### 3.4 A Study on the Adhesion of Surface Protein to Three Kinds of Cells

**FIGURE 5:**
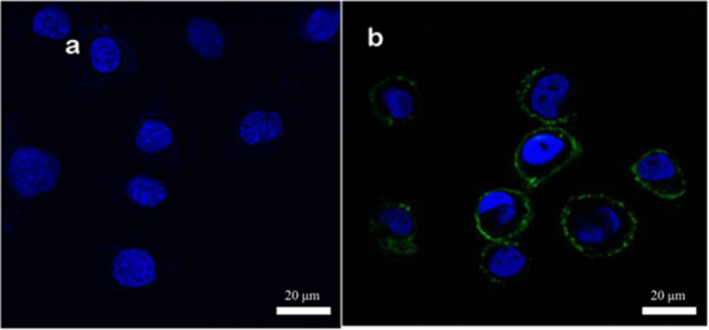
Adhesion of surface protein to Caco‐2 cells. (a) Control group. Caco‐2 cells without surface protein were added with DAPI and fluorescence photos were taken. (b) Experimental group. Caco‐2 cells with surface protein were added with DAPI and fluorescence photos were taken. The surface protein concentration was 1.59 g/L.

**FIGURE 6:**
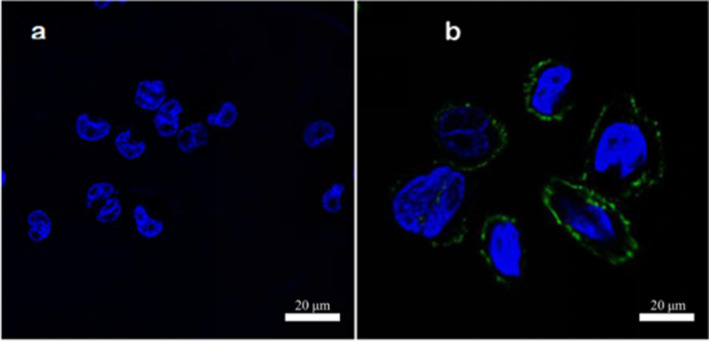
Adhesion of surface protein to mouse colon cancer cells. (a) Control group, and the mouse colon cancer cells without surface protein were added with DAPI and then fluorescence photographed. (b) Experimental group, and the mouse colon cancer cells with surface protein were added with DAPI and then fluorescence photographed, and the surface protein concentration was 1.59 g/L.

**FIGURE 7:**
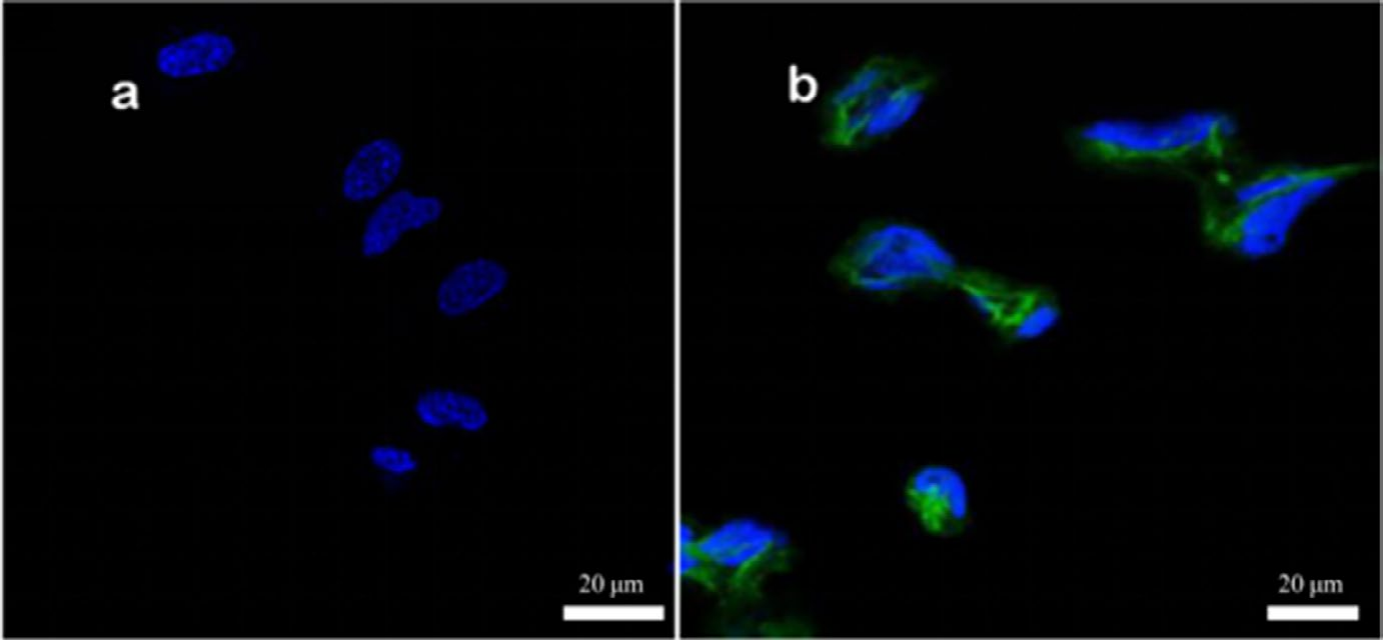
Adhesion of surface protein to chicken intestinal epithelial cells. (a) Control group, and the chicken intestinal epithelial mucosa cells without surface protein were added with DAPI for fluorescence photography. (b) Experimental group, and the chicken intestinal epithelial mucosa cells with surface protein were added with DAPI for fluorescence photography, and the surface protein concentration was 1.59 g/L.

In the experiment, surface proteins of medium concentration (1.59 g/L) were labeled with fluorescent FITC. Adhesion experiments were conducted separately on Caco‐2 cells, mouse colon cancer cells, and chicken intestinal epithelial cells. Upon magnification by confocal microscopy at 630×, the results from Figures 5, 6, 7 reveal that after incubating the three cell types with the same concentration of surface proteins, the fluorescence ring thickness of Caco‐2 cells and mouse colon cancer cells is similar. This suggests that the fluorescence intensity generated by surface proteins is comparable but lower than that observed in chicken intestinal epithelial cells. The findings indicate that surface proteins adsorb most prominently to the surface of chicken intestinal epithelial cells.

### 3.5 Inhibition of Salmonella Adhesion by Surface Protein

#### 3.5.1 Adhesion of Different Concentrations of Surface Protein to Chicken Intestinal Mucosa Cells

**FIGURE 8:**
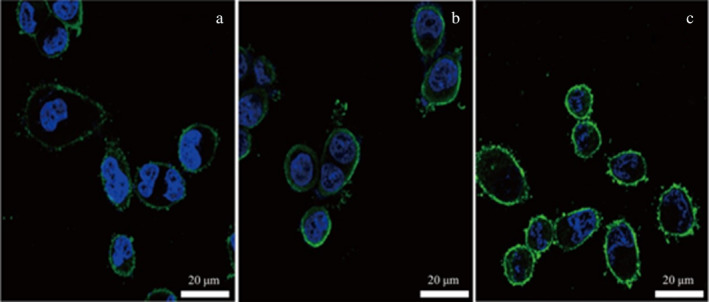
Adhesion of different concentrations of surface protein to chicken intestinal epithelial cells. (a) Fluorescence microscope combined image of low concentration of surface protein adhering to chicken intestinal epithelial mucosa cells. (b) Fluorescence microscope combined image of medium concentration of surface protein adhering to chicken intestinal epithelial mucosa cells. (c) Fluorescence microscope combined image of high concentration of surface protein adhering to chicken intestinal epithelial mucosa cells.

In this experiment, surface proteins were initially divided into three different concentrations (0.84, 1.59, and 2.61 g/L) and then labeled with FITC dye. The labeled surface proteins were added to well‐cultured chicken intestinal epithelial cells for incubation. The adhesion of surface proteins to chicken intestinal mucosal cells was observed under a fluorescence microscope after adding different concentrations of surface proteins. Figure 8 depicts the adhesion of chicken intestinal mucosal cells after the addition of low, medium, and high concentrations of surface proteins. From Figure 8, it is evident that the addition of high‐concentration surface proteins results in the strongest fluorescence intensity on the surface of chicken intestinal mucosal cells, indicating that surface proteins exhibit the highest adhesion to chicken intestinal mucosal cells.

#### 3.5.2 Results of Inhibition of Salmonella Adhesion to Chicken Intestinal Mucosal Cells by Surface Proteins of Different Concentrations

**FIGURE 9:**
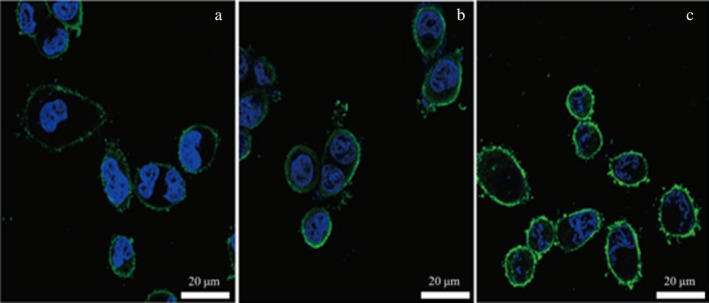
Inhibition of Salmonella adhesion to chicken intestinal epithelial cells after addition of different concentrations of surface protein. (a) Fluorescence microscope combined image of inhibition of Salmonella adhesion to chicken intestinal mucosal epithelial cells after addition of low concentration of surface protein. (b) Fluorescence microscope combined image of inhibition of Salmonella adhesion to chicken intestinal mucosal epithelial cells after addition of medium concentration of surface protein. (c) Fluorescence microscope combined image of inhibition of Salmonella adhesion to chicken intestinal mucosal epithelial cells after addition of high concentration of surface protein.

In this experiment, surface proteins were divided into three different concentrations and labeled with FITC dye. The labeled surface proteins were added to chicken intestinal mucosal cells for incubation. The adhesion of Salmonella to chicken intestinal mucosa was observed under a fluorescence microscope after adding different concentrations of surface proteins. Fluorescence intensity was quantified using a fluorescence spectrometer and expressed in arbitrary units (AU). Figure 9 illustrates the specific details of surface protein inhibition of Salmonella adhesion to chicken intestinal mucosal cells after the addition of low, medium, and high concentrations of surface proteins. Table 2 presents the results of plate counting to detect the adhesion of Salmonella to the surface of chicken intestinal mucosal cells.

Combining Figure 9 and Table 2, it can be observed that adding a low concentration of surface proteins resulted in a Salmonella concentration of 10^8^ CFU/mL, as a large number of Salmonella competed to adhere to the chicken intestinal mucosal surface. The plate counting results showed a Salmonella adhesion quantity of 7.20 × 10^6^ CFU/mL, leading to the lowest fluorescence intensity of surface proteins adhering to the chicken intestinal mucosal surface (fluorescence intensity: 15 AU). In the medium concentration of surface proteins, more surface proteins participated in inhibiting Salmonella adhesion to chicken intestinal mucosa. The plate counting results revealed a reduced Salmonella adhesion quantity of 3.68 × 10^3^ CFU/mL, with an increased fluorescence intensity of surface proteins adhering to the cell surface (fluorescence intensity: 45 AU). The addition of high‐concentration surface proteins resulted in the strongest fluorescence intensity (fluorescence intensity: 90 AU), and plate counting indicated a reduced Salmonella adhesion quantity to 0 CFU/mL, demonstrating the most effective inhibition of Salmonella adhesion to chicken intestinal mucosal cells. This indirectly confirms the inhibitory effect of the added surface proteins on Salmonella adhesion. Previous research has confirmed that *L*. *acidophilus* ATCC 4356 can reduce the adhesion and invasion of pathogenic bacteria to intestinal epithelial cells through the competitive action of surface proteins (Chen et al. 2007b). The surface proteins of *L*. *crispatus* ZJ001 also exhibit competitive inhibitory effects on the adhesion of *S*. *typhimurium* and *E*. *coli* 0157:H7 to HeLa cells (Gay and Keith 1991), aligning with the findings of this experiment (Table 4).

**TABLE 4:** Inhibition of *Salmonella* adhesion cells by different concentrations of surface protein.

Treatment groups	Salmonella + Lactobacillus adherent cells	Salmonella plate count
	Surface protein‐a + SC79	7.20 × l0^6^ ± 0.67 CFU/mL
Experimental group	Surface protein‐b + SC79	3.68 × l0^3^ ± 0.49 CFU/mL
	Surface protein‐c + SC79	0
Control group	SC79 without surface protein	3.79 × l0^8^ ± 0.33 CFU/mL

*Note:* a: Low concentration surface protein 0.84 g/L, b: medium concentration surface protein 1.59 g/L, c: high concentration surface protein 2.61 g/L.

#### 3.5.3 Inhibitory Effect of Surface Protein on Salmonella After Adding Different Concentrations of Lactic Acid Bacteria

**FIGURE 10:**
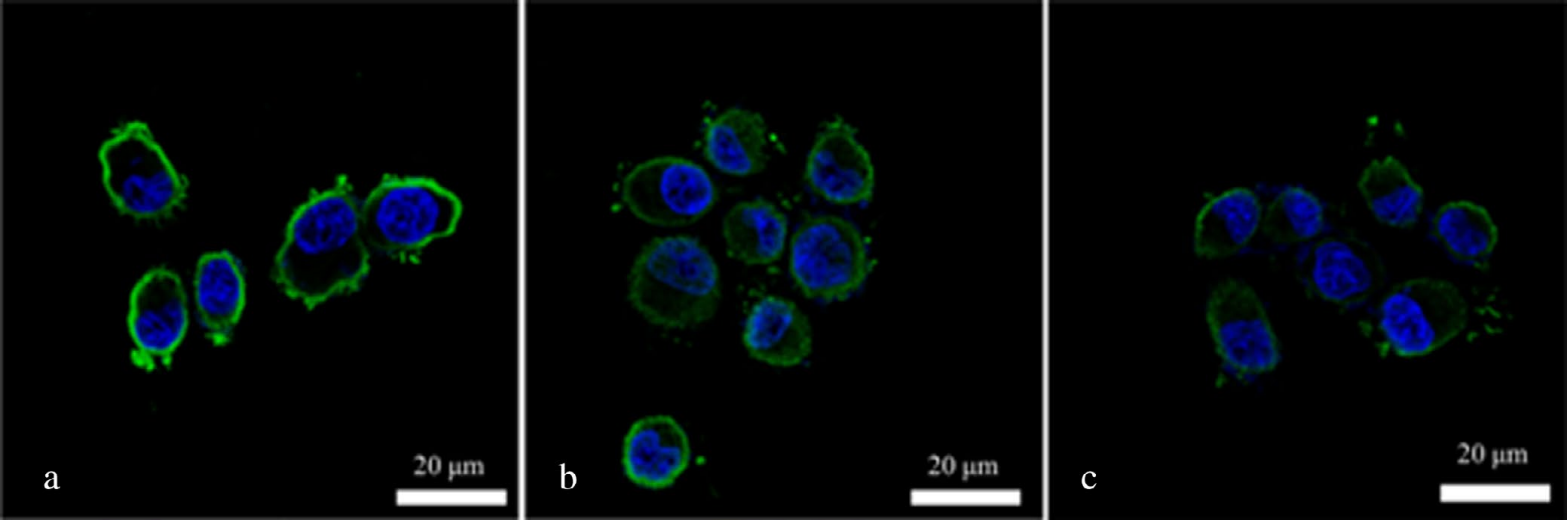
Inhibition of Salmonella adhesion to chicken intestinal epithelial cells by the addition of surface protein and different concentrations of LAB69. (A) Adding low concentration lactic acid bacteria (1 × 10^2^ CFU/mL). (B) Medium concentration of lactic acid bacteria added in the figure (1 × 10^5^ CFU/mL). (C) Combined fluorescence microscope image of inhibiting Salmonella adhesion to chicken intestinal epithelial cells after adding lactic acid bacteria at high concentration (1 × 10^8^ CFU/mL), with the surface protein concentration of 2.61 g/L.

In this experiment, the concentration of surface proteins was initially adjusted to 2.61 g/L and then labeled with FITC dye. The labeled surface proteins were added to well‐cultured chicken intestinal mucosal cells for incubation. Using confocal microscopy, the adhesion of Salmonella to chicken intestinal mucosa was observed after adding different concentrations of LAB69 probiotics, and the quantity of Salmonella was determined by plate counting. Figure 10 presents the confocal microscopy results of Salmonella adhesion to chicken intestinal mucosal cells after the addition of low, medium, and high concentrations of LAB69 probiotics.

In the case of low‐concentration LAB69 probiotics, there was inhibition of Salmonella, with a large amount of surface proteins adhering to chicken intestinal mucosal cells, resulting in the strongest fluorescence intensity. After the addition of medium‐concentration LAB69 probiotics, the fluorescence intensity decreased, suggesting that surface proteins enhanced the ability of LAB69 probiotics to inhibit Salmonella, leading to the participation of some LAB69 probiotics in adhering to chicken intestinal cells and causing a decrease in fluorescence intensity. With the addition of high‐concentration LAB69 probiotics, the surface fluorescence intensity was the weakest. It is speculated that a large quantity of LAB69 probiotics adhered to chicken intestinal mucosal cells, indicating that high‐concentration LAB69 probiotics antagonized Salmonella adhesion to chicken intestinal mucosal cells. The excess LAB69 probiotics adhered to the surface of chicken intestinal mucosal cells, reducing the adhesion of surface proteins to chicken intestinal mucosal cells and causing a decrease in fluorescence intensity.

## 4 Cytokine Expression Before and After Adding Surface Protein in Three Kinds of Cells Incubated With Lactic Acid Bacteria

In the experiment, the control group (CK) involved incubating Caco‐2 cells, mouse colon cancer cells, and chicken intestinal mucosal cells in PBS for 2 h. Subsequently, RNA was extracted, and the expression of cytokines was assessed. Treatment group 1 included incubation with *Lactobacillus salinus* strain HO 66 (LAB35) without extracted surface proteins, *Lactobacillus* sp. wx213 (LAB69) with a high surface protein content, and the standard strain *L*. *rhamnosus G* (LG, FSMM22) for 2 h with three cell types (Caco‐2 cells, mouse colon cancer cells, and chicken intestinal mucosal cells). RNA was then extracted, and cytokine expression was evaluated. Treatment group 2 involved incubation with LAB35, LAB69, and LG, with the addition of surface proteins extracted from LAB69 (concentration of 1.59 g/L), followed by 2 h of incubation with three cell types. RNA was extracted, and cytokine expression was assessed.

**FIGURE 11:**
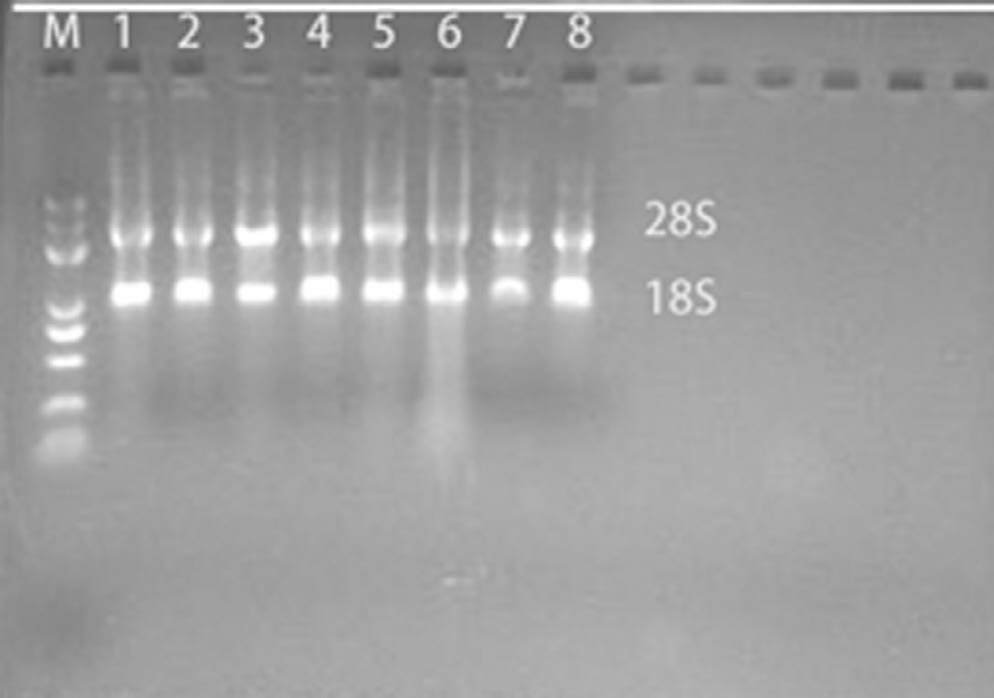
Denatured gel electrophoresis of RNA extracted from different bacteria treatment groups. M is Mark, Line1 is CK treatment group, Line2 is LAB31 treatment group, Line3 is LAB35 treatment group, Line4 is LAB69 treatment group, Line5 is L76 treatment group, Line6 is LG treatment group, Line7 is SC79 treatment group, Line8 is SE05 treatment group.

The extracted RNA had a concentration of ~1000 ng/μL. Denaturing gel electrophoresis revealed clear bands at 28 and 18 s, indicating minimal impurities in the extracted RNA, making it suitable for further experiments. β‐Actin was used as a commonly used internal reference in PCR. By analyzing the Ct values of the target gene amplification curve and normalizing to the Ct value of the β‐actin internal reference amplification curve, background template differences could be eliminated. This allowed the quantification of mRNA expression levels of the target gene influenced by different treatments with Lactobacillus and Salmonella isolates. The internal reference gene β‐actin exhibited similar Ct values during PCR amplification, suggesting comparable template DNA concentrations during amplification (Figure 11).

### 4.1 Cytokine Myeloid Differentiation Protein 2 Expression

**FIGURE 12:**
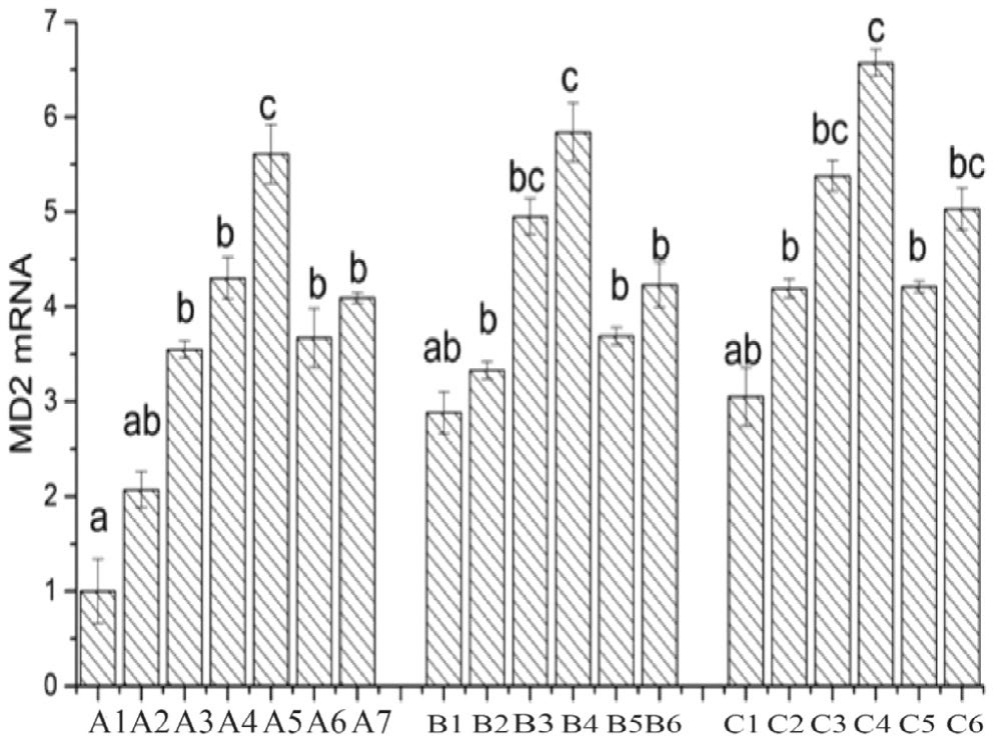
Expression of MD2 in Lactobacillus before and after adding surface protein. A1: CK; A2: LAB35‐Caco‐2; A3: LAB35 + S‐Caco‐2; A4: LAB69‐Caco‐2; A5: LAB69 + S‐Caco‐2; A6: LG‐Caco‐2; A7: LG + S‐Caco‐2; B1: LAB35‐CT26.WT; B2: LAB35 + S‐CT26.WT; B3: LAB69‐CT26.WT; B4: LAB69 + S‐CT26.WT; B5: LG‐CT26.WT; B6: LG + S‐CT26.WT; C1: LAB35‐CHI‐iCell; C2: LAB35 + S‐CHI‐iCell; C3: LAB69‐CHI‐iCell; C4: LAB69 + S‐CHI‐iCell; C5: LG‐CHI‐iCell; C6: LG + S‐CHI‐iCell. There were no statistically significant differences in common superscript letters (*p* < 0.05).

MD2 is a secreted protein expressed on the cell membrane, and its function was initially discovered by Shimazu et al. (1999). MD2 binds to the extracellular domain of TLR4 and exhibits a physiological correlation with TLR4. As shown in Figure 12, cells incubated with PBS represent the CK group, with the mRNA expression of the MD2 gene in the CK group set as 1. Compared to the CK group, the mRNA expression of the MD2 gene is upregulated in all other groups after cell treatment.

In Caco‐2 cells incubated with lactic acid bacteria alone, the mRNA expression of the MD2 gene follows the order: LAB69 > LG > LAB35. After the addition of surface proteins to *Lactobacillus* LAB35 and LAB69, the mRNA expression of the MD2 gene is significantly upregulated (compared to ‐‐) (*p* < 0.01). In the mouse colon cancer cell group, there is a difference in the expression level of the MD2 gene after the addition of surface proteins, and only the mRNA expression of the MD2 gene in LAB69 is significantly upregulated (*p* < 0.01). In the chicken intestinal mucosal epithelial cell group, the mRNA expression of the MD2 gene in LAB35 and LAB69 is significantly upregulated (*p* < 0.01), and LG's MD2 gene expression is also upregulated (0.01 < *p* < 0.05).

### 4.2 Expression of the Cytokine Toll Receptor 4

**FIGURE 13:**
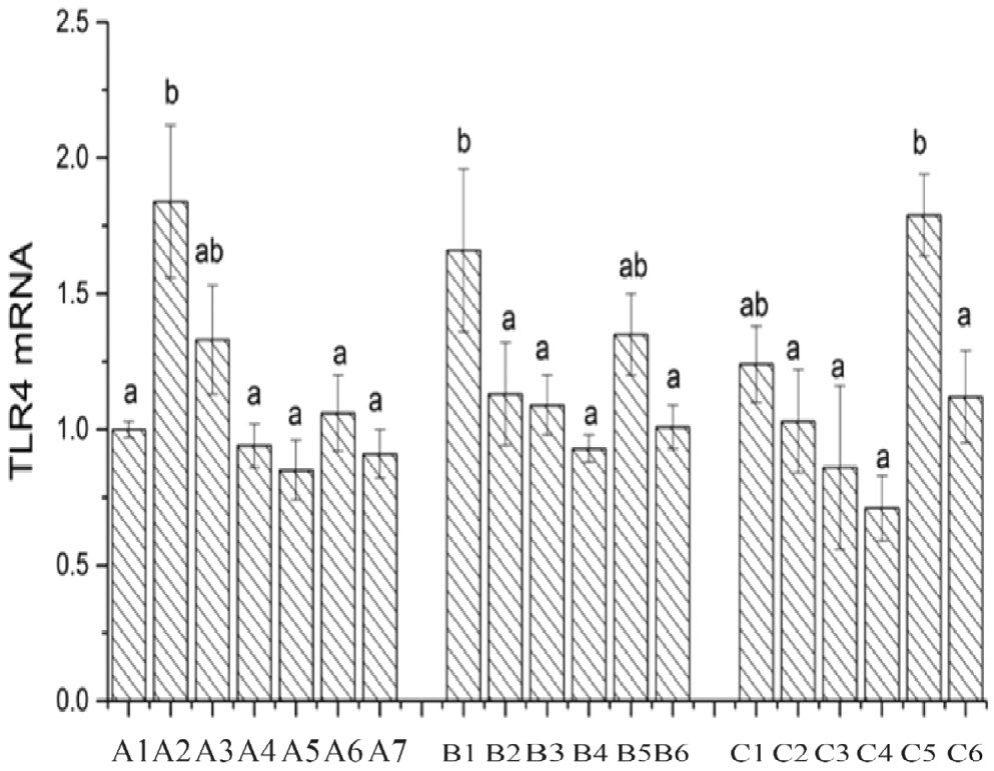
Expression of TLR4 in Lactobacillus before and after adding surface protein. A1: CK; A2: LAB35‐Caco‐2; A3: LAB35 + S‐Caco‐2; A4: LAB69‐Caco‐2; A5: LAB69 + S‐Caco‐2; A6: LG‐Caco‐2; A7: LG + S‐Caco‐2. B1: LAB35‐CT26.WT; B2: LAB35 + S‐CT26.WT; B3: LAB69‐CT26.WT; B4: LAB69 + S‐CT26.WT; B5: LG‐CT26.WT; B6: LG + S‐CT26.WT. C1: LAB35‐CHI‐iCell; C2: LAB35 + S‐CHI‐iCell; C3: LAB69‐CHI‐iCell; C4: LAB69 + S‐CHI‐iCell; C5: LG‐ CHI‐iCell; C6: LG + S‐CHI‐iCell. There were no statistically significant differences in common superscript letters (*p* < 0.05).

TLR4 is a crucial protein molecule involved in the host's innate immunity and serves as a vital link between innate and adaptive immunity (Hymes et al. 2016). After bacteria breach the physical barriers of the host, such as the skin and mucous membranes, TLR recognizes molecules with conserved structures in foreign microorganisms, activating the immune cells to initiate an inflammatory response (Shekels et al. 2001). As observed in Figure 13, the expression of the TLR4 gene in the CK group is set as 1. In comparison to the CK group, the expression of the TLR4 gene is upregulated in chicken‐derived Lactobacillus LAB35‐treated cells, whereas downregulated in chicken‐derived Lactobacillus LAB69 and the standard strain LG. After the addition of surface proteins, the expression of the TLR4 gene is downregulated in all treatment groups, with the expression in chicken‐derived Lactobacillus LAB35 higher than that in Lactobacillus LAB69 and the standard strain LG. Across three different host cells, it can be observed that the expression of the TLR4 gene in chicken‐derived Lactobacillus LAB35 and LAB69‐treated chicken intestinal mucosal cells is downregulated compared to mouse colon cancer cells and Caco‐2 cells. In contrast, the expression of the TLR4 gene in the standard strain LG is upregulated, possibly due to the fact that chicken‐derived Lactobacillus belongs to the same population as chicken intestinal mucosal cells, whereas the standard strain LG is from a different population.

### 4.3 Expression of the Cytokine Interleukin 10

**FIGURE 14:**
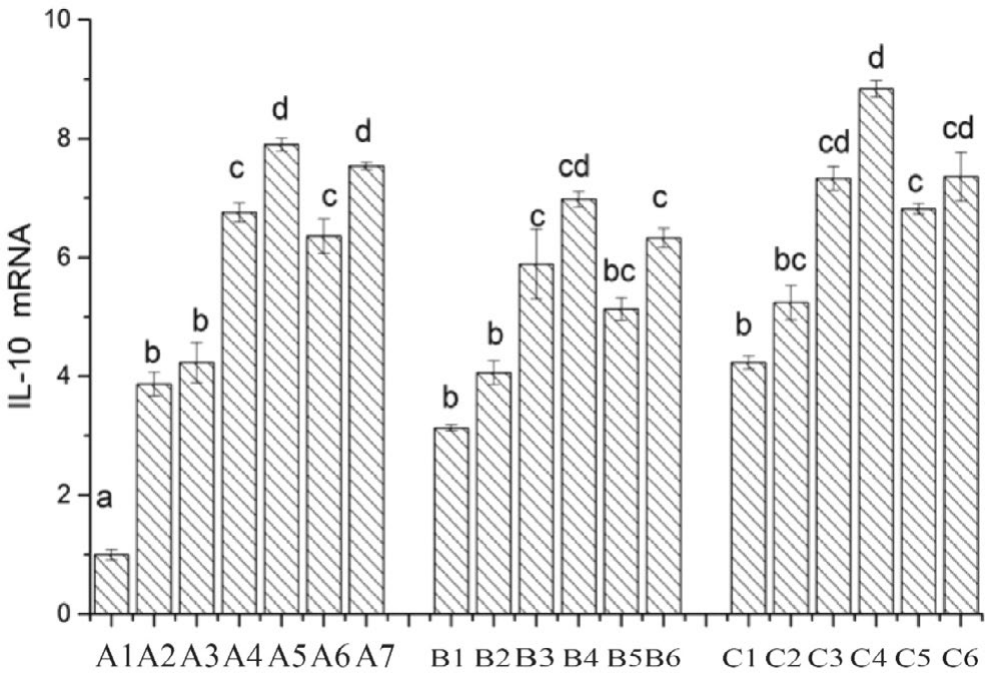
Expression of IL‐10 in Lactobacillus before and after adding surface protein. A1: CK; A2: LAB35‐Caco‐2; A3: LAB35 + S‐Caco‐2; A4: LAB69‐Caco‐2; A5: LAB69 + S‐Caco‐2; A6: LG‐Caco‐2; A7: LG + S‐Caco‐2. B1: LAB35‐CT26.WT; B2: LAB35 + S‐CT26.WT; B3: LAB69‐CT26.WT; B4: LAB69 + S‐CT26.WT; B5: LG‐CT26.WT; B6: LG + S‐CT26.WT. C1: LAB35‐CHI‐iCell; C2: LAB35 + S‐CHI‐iCell; C3: LAB69‐CHI‐iCell; C4: LAB69 + S‐CHI‐iCell; C5: LG‐ CHI‐iCell; C6: LG + S‐CHI‐iCell. The differences without common superscript letters were statistically significant (*p* < 0.05).

Interleukin‐10 (IL‐10) is a versatile and multi‐cellular cytokine that regulates cell growth and differentiation, participating in both inflammatory and immune responses. It is widely recognized as an anti‐inflammatory and immunosuppressive factor (Ho et al. 2010). As depicted in Figure 14, the mRNA expression of the IL‐10 gene in the CK group is set as 1. In comparison to the CK group, the mRNA expression of the IL‐10 gene is upregulated in all other treatment groups. When individually cultivating Caco‐2 cells with Lactobacillus, the mRNA expression of the IL‐10 gene follows the order LAB69 > LG > LAB35. After the addition of surface proteins, the change in expression of the IL‐10 gene in Lactobacillus LAB35 is not significant (*p* > 0.05), whereas it is significantly upregulated in Lactobacillus LAB69 and the standard strain LG (0.01 < *p* < 0.05). In the mouse colon cancer cell group, after the addition of surface proteins, the IL‐10 gene expression is significantly upregulated in LAB35, LAB69, and LG (0.01 < *p* < 0.05). In the chicken intestinal mucosal epithelial cell group, the IL‐10 gene expression is significantly upregulated in LAB69 (*p* < 0.01), and in LAB35, the IL‐10 gene expression is also significantly upregulated (0.01 < *p* < 0.05).

### 4.4 Expression of Mucin 3

**FIGURE 15:**
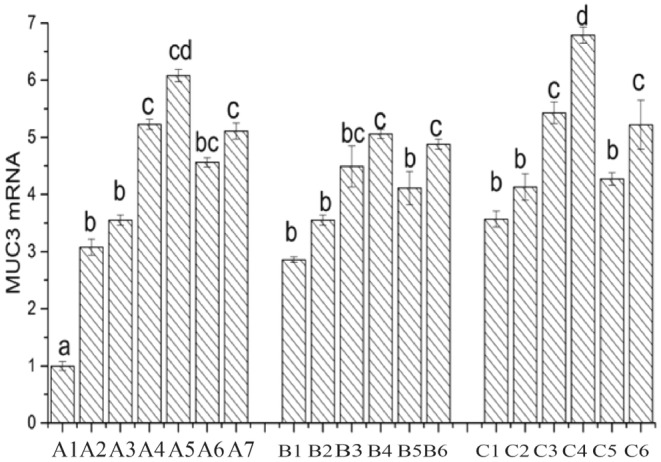
Expression of MUC3 in Lactobacillus before and after adding surface protein. A1: CK; A2: LAB35‐Caco‐2; A3: LAB35 + S‐Caco‐2; A4: LAB69‐Caco‐2; A5: LAB69 + S‐Caco‐2; A6: LG‐Caco‐2; A7: LG + S‐Caco‐2. B1: LAB35‐CT26.WT; B2: LAB35 + S‐CT26.WT; B3: LAB69‐CT26.WT; B4: LAB69 + S‐CT26.WT; B5: LG‐CT26.WT; B6: LG + S‐CT26.WT. C1: LAB35‐CHI‐iCell; C2: LAB35 + S‐CHI‐iCell; C3: LAB69‐CHI‐iCell; C4: LAB69 + S‐CHI‐iCell; C5: LG‐CHI‐iCell; C6: LG + S‐CHI‐iCell. There was no statistically significant difference in common superscript letters (*p* < 0.05).

As a typical transmembrane mucin, MUC3 is predominantly expressed in intestinal cells. Numerous studies have confirmed the protective role of MUC3 in the intestinal mucosa (El et al. 2007). As shown in Figure 15, the mRNA expression of the MUC3 gene in the CK group is set as 1. In comparison to the CK group, the mRNA expression of the MUC3 gene is significantly upregulated in all other treatment groups. When individually cultivating Caco‐2 cells with Lactobacillus, the mRNA expression of the MUC3 gene follows the order LAB69 > LG > LAB35. After the addition of surface proteins in Lactobacillus LAB69, the mRNA expression of the MUC3 gene is significantly upregulated (0.01 < *p* < 0.05), and in Lactobacillus LAB35 and the standard strain LG, the mRNA expression of the MUC3 gene is also upregulated (*p* < 0.05). In the mouse colon cancer cell group, after the addition of surface proteins, the MUC3 gene expression is significantly upregulated in LAB35 and LG (0.01 < *p* < 0.05). In the chicken intestinal mucosal epithelial cell group, the MUC3 gene expression is significantly upregulated in LAB69 (*p* < 0.01), and in LG, the MUC3 gene expression is also significantly upregulated (0.01 < *p* < 0.05).

### 4.5 Cytokines Macrophage Autocrine Factor Expression

**FIGURE 16:**
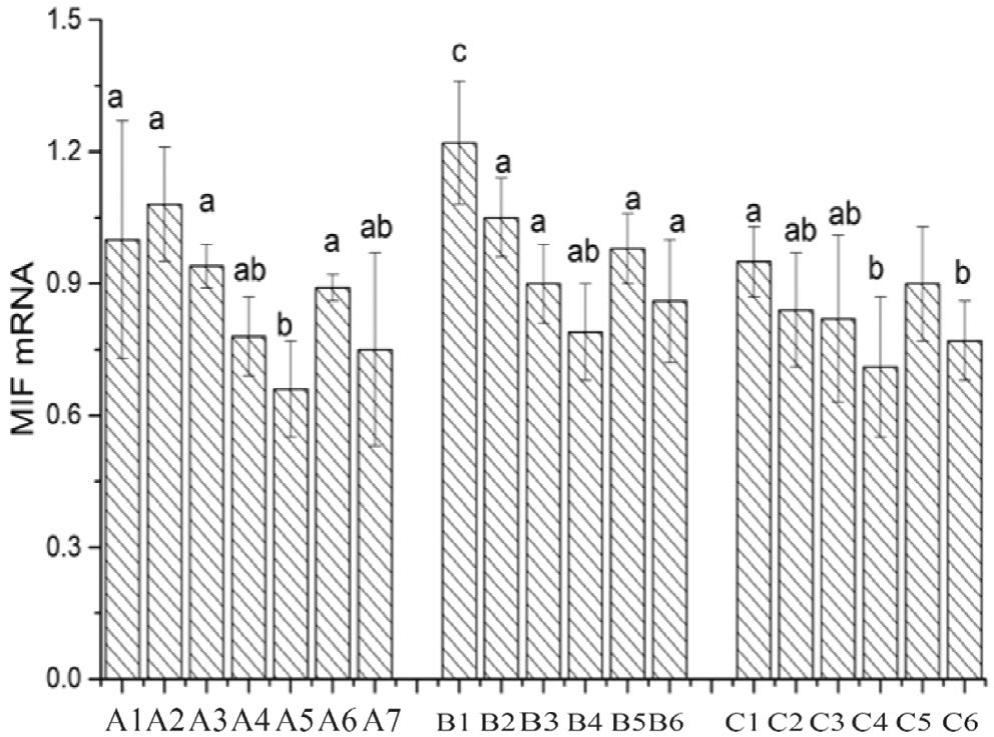
Expression of MIF in Lactobacillus before and after adding surface protein. A1: CK; A2: LAB35‐Caco‐2; A3: LAB35 + S‐Caco‐2; A4: LAB69‐Caco‐2; A5: LAB69 + S‐Caco‐2; A6: LG‐Caco‐2; A7: LG + S‐Caco‐2. B1: LAB35‐CT26.WT; B2: LAB35 + S‐CT26.WT; B3: LAB69‐CT26.WT; B4: LAB69 + S‐CT26.WT; B5: LG‐CT26.WT; B6: LG + S‐CT26.WT. C1: LAB35‐CHI‐iCell; C2: LAB35 + S‐CHI‐iCell; C3: LAB69‐CHI‐iCell; C4: LAB69 + S‐CHI‐iCell; C5: LG‐ CHI‐iCell; C6: LG + S‐CHI‐iCell. There was no statistically significant difference in common superscript letters (*p* < 0.05).

Macrophage migration inhibitory factor (MIF) is an autocrine factor secreted by macrophages (Ting and Guihua 2004). It regulates the expression of host cytokines through both innate and acquired immune responses, enhancing macrophage adhesion and phagocytosis. After infection with Salmonella, MIF promotes the expression of inflammatory factors such as IL‐6, IL‐8, TNF‐α, and INF‐γ, thereby strengthening the functional role of macrophages (Ren et al. 2004). As shown in Figure 16, the mRNA expression of the MIF gene in the CK group is set as 1. In comparison to the CK group, except for the individual cultivation of Caco‐2 cells with Lactobacillus LAB35, the mRNA expression of the MIF gene is downregulated in all other treatment groups. When individually cultivating Caco‐2 cells with Lactobacillus, the mRNA expression of the MIF gene follows the order LAB69 < LG < LAB35. After the addition of surface proteins in the three Lactobacillus strains, the mRNA expression of the MIF gene is significantly downregulated (0.01 < *p* < 0.05). In the mouse colon cancer cell group, after the addition of surface proteins, the MIF gene expression in the three Lactobacillus strains is significantly downregulated (0.01 < *p* < 0.05). In the chicken intestinal mucosal epithelial cell group, after the addition of surface proteins, the MIF gene expression in the three Lactobacillus strains is significantly downregulated (0.01 < *p* < 0.05).

### 4.6 Expression of Inflammatory Factor Tumor Necrosis Factor Alpha

When the host is invaded by pathogenic bacteria, Toll‐like receptors (TLRs) transmit inflammatory signals to the immune system, leading to the production of inflammatory factors. This stimulates the production of Macrophage Migration Inhibitory Factor (MIF), which prevents excessive phagocytosis of macrophages. In 1975, Carswell discovered a factor that could kill tumor cells, named tumor necrosis factor (TNF) (Carswell et al. 1975). Subsequent research by Shalaby named the TNF produced by macrophages as TNF‐α. TNF‐α has anti‐infective capabilities and is an important inflammatory factor (Shalaby et al. 1995).

**FIGURE 17:**
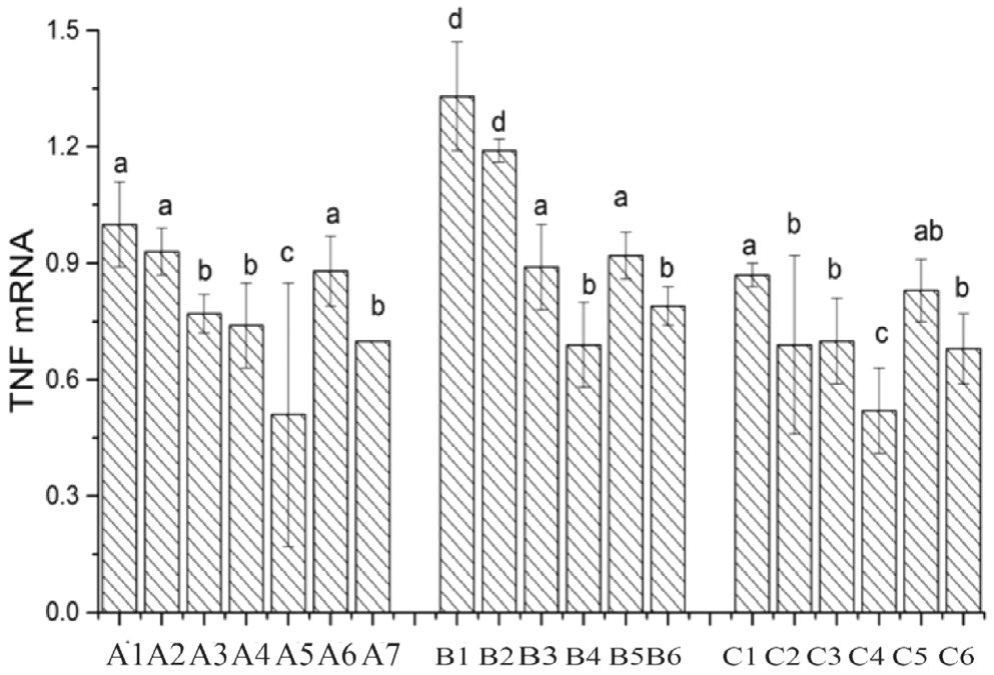
Expression of TNF‐α in Lactobacillus before and after adding surface protein. A1: CK; A2: LAB35‐Caco‐2; A3: LAB35 + S‐Caco‐2; A4: LAB69‐Caco‐2; A5: LAB69 + S‐Caco‐2; A6: LG‐Caco‐2; A7: LG + S‐Caco‐2. B1: LAB35‐CT26.WT; B2: LAB35 + S‐CT26.WT; B3: LAB69‐CT26.WT; B4: LAB69 + S‐CT26.WT; B5: LG‐CT26.WT; B6: LG + S‐CT26.WT. C1: LAB35‐CHI‐iCell; C2: LAB35 + S‐CHI‐iCell; C3: LAB69‐CHI‐iCell; C4: LAB69 + S‐CHI‐iCell; C5: LG‐ CHI‐iCell; C6: LG + S‐CHI‐iCell. The difference without common superscript letters was statistically significant (*p* < 0.01).

As shown in Figure 17, the mRNA expression of the TNF‐α gene in the CK group is set as 1. In comparison to the CK group, except for the individual cultivation of mouse colon cancer cells with Lactobacillus LAB35, the mRNA expression of the TNF‐α gene is downregulated in all other treatment groups. When individually cultivating Caco‐2 cells with Lactobacillus, the mRNA expression of the TNF‐α gene follows the order LAB69 < LG < LAB35. After the addition of surface proteins, the mRNA expression of the TNF‐α gene is significantly downregulated for Lactobacillus LAB35 and LG (0.01 < *p* < 0.05), and for Lactobacillus LAB69, it is significantly downregulated (*p* < 0.01). In the mouse colon cancer cell group, after the addition of surface proteins, the mRNA expression of the TNF‐α gene in Lactobacillus LAB69 is significantly downregulated (*p* < 0.01). In the chicken intestinal mucosal epithelial cell group, after the addition of surface proteins, the expression of the TNF‐α gene in the three Lactobacillus strains is significantly downregulated (0.01 < *p* < 0.05).

Research has revealed that the surface proteins of lactic acid bacteria, upon binding to receptors on intestinal epithelial cells, can stimulate the proliferation and maturation of phagocytes and lymphocytes, thereby enhancing both humoral and cellular immune functions within the host (Kawase et al. 2012). However, due to the intricate composition of lactic acid bacteria surface components, there is still a need for further investigation into the constituents and immunomodulatory functions of lactic acid bacteria surface proteins in their interaction with immune cells. In 2016, Haihong, Zhenquan, and Xiaolin (2016) explored the effects of surface proteins from five different strains of lactic acid bacteria on three antigen‐presenting cell types. Their analysis of the impact of surface proteins on adhesion and inducement of proliferative effects confirmed that these proteins mediate the attachment of lactic acid bacteria to target sites on cells, thereby regulating the growth and metabolic pathways of immune cells. The findings from this experiment corroborate the significance of lactic acid bacteria surface proteins as a crucial foundation for adhesion and induction of immune cell proliferation. This serves as a basis for further identification of immunomodulatory molecules within lactic acid bacteria surface proteins and the scientific application of microbial ecological agents.

## 5 Discusses

These results provide a comprehensive understanding of the adhesion behavior of LAB strains to different cell types. The observed reduction in bacterial adhesion following the addition of surface proteins suggests potential competitive interactions at the cell surface. This lays the groundwork for further exploration into the specific molecular mechanisms involved.

In the experiment, surface proteins were labeled with FITC and used in adhesion assays with Caco‐2 cells, mouse colon cancer cells, and chicken intestinal epithelial mucosal cells. Using the confocal function of an inverted fluorescence microscope, the adhesion of the proteins to the three types of cells was compared. The results showed that the fluorescence intensity on the surface of chicken intestinal mucosal cells was higher than that of the other two cell types, indicating that the surface proteins adhered most to the chicken intestinal mucosal cells.

Yingxue et al. (2020) demonstrated that FITC‐labeled *Lactobacillus acidophilus* adhered to HT‐29 cells, showing that S‐layer proteins participated in the adhesion of *Lactobacillus acidophilus* to intestinal epithelial cells. When the S‐layer proteins were removed, the adhesion of *Lactobacillus acidophilus* to intestinal cells significantly decreased. This finding is consistent with some of our experimental results. Prado Acosta et al. (2019) noted that surface proteins of lactobacilli could inhibit the adhesion of pathogenic bacteria by binding to cell surface receptors.

In the experiment, labeled surface proteins at different concentrations were added to cultured chicken intestinal mucosal cells and incubated. The adhesion of Salmonella to the chicken intestinal mucosal cells was observed under a fluorescence microscope. The addition of high concentrations of surface proteins resulted in the brightest fluorescence on the chicken intestinal mucosal cells, indicating the highest fluorescence intensity and maximal adhesion of surface proteins. This indirectly proved that the addition of surface proteins inhibited the adhesion of Salmonella to the chicken intestinal mucosal cells. When different concentrations of LAB69 were added to the cultured chicken intestinal mucosal cells, the adhesion of *Salmonella* was also observed. With low and medium concentrations of LAB69, the fluorescence on the chicken intestinal mucosal cells was relatively bright, indicating higher fluorescence intensity and more surface protein adhesion. This suggested that lower concentrations of LAB69 inhibited *Salmonella* adhesion, allowing more surface proteins to adhere to the cell surface. In contrast, the addition of high concentrations of LAB69 resulted in dimmer fluorescence, indicating weaker fluorescence intensity and less surface protein adhesion. This may be due to the lactobacilli partially adhering to the chicken intestinal mucosal cells, reducing the adhesion rate of the surface proteins.

Comparing the results of different concentrations of lactobacilli inhibiting Salmonella adhesion to the positive control of Salmonella adhesion to chicken intestinal mucosal cells, it was found that the addition of lactobacilli reduced *Salmonella* adhesion. The higher the concentration of lactobacilli, the better the inhibitory effect.

S‐layer proteins have the functions of repairing damaged intestinal cells, regulating cell signaling pathways, and modulating the secretion of inflammatory factors (Rask et al. 2013). Our experiment measured cytokines and found that the incubation of the three types of cells with lactobacilli supplemented with surface proteins enhanced the host cell's inflammatory response and increased the body's anti‐infection ability, consistent with Li Honghai's experimental results (Haihong, Zhenquan, and Xiaolin 2016). Under normal conditions, TLR4 expression is very low to maintain homeostasis and balance (Abreu et al. 2001). Observation of the results showed that the addition of surface proteins downregulated TLR4 expression. The analysis of the gene expression of the inflammatory cytokine TNF‐α revealed that the mRNA expression of TNF‐α was significantly downregulated by LAB35 and LG (0.01 < *p* < 0.05) and significantly downregulated by LAB69 (*p* < 0.01). In the mouse colon cancer cell group, the mRNA expression of TNF‐α was significantly downregulated by LAB69 after the addition of surface proteins (*p* < 0.001). In the chicken intestinal mucosal epithelial cell group, the TNF‐α gene expression was significantly downregulated by all three strains of lactobacilli after the addition of surface proteins (0.01 < *p* < 0.05).

Friis, Monika, and Taylor (2009) found that S‐layer proteins could block MyD88 pathway signal transduction in Caco‐2 cells, thereby inhibiting Salmonella‐induced IL‐6 secretion, mediated through Toll‐like receptor (TLR‐2). Konstantinov et al. (2008) discovered that during the interaction between *Lactobacillus acidophilus* NCF and dendritic cells, S‐layer proteins induced the synthesis and secretion of IL‐10 and IL‐12, consistent with our research findings.

## Author Contributions

**Ziheng Meng:** data curation (equal), validation (equal), writing – original draft (equal). **Xianqing Huang:** conceptualization (equal), funding acquisition (equal). **Mingwu Qiao:** conceptualization (equal), visualization (equal). **Lianjun Song:** project administration (equal), supervision (equal). **Yufei Liu:** investigation (equal), methodology (equal). **Dan Hai:** data curation (equal), resources (equal), software (equal).

## Ethics Statement

This study does not involve any human or animal testing.

## Consent

Written informed consent was obtained from all study participants.

## Conflicts of Interest

The authors declare no conflicts of interest.

## Data Availability

The authors will supply the relevant data in response to reasonable requests.
